# Dietary inflammatory index in relation to incident CKD: A prospective study of UK Biobank participants

**DOI:** 10.1371/journal.pone.0341502

**Published:** 2026-02-20

**Authors:** Qian Yang, Queran Lin, Yixi Liu, Pufei Bai, Suhua Gao, Xin Lv, Saijun Zhou, Hongyan Liu, HaiZhen Sun, Pei Yu

**Affiliations:** 1 NHC Key Lab of Hormones and Development and Tianjin Key Lab of Metabolic Diseases, Tianjin Medical University Chu Hsien-I Memorial Hospital & Institute of Endocrinology, Tianjin, China; 2 Breast Tumor Center, Sun Yat-sen Memorial Hospital, Sun Yat-sen University, Guangzhou, China; 3 Department of Primary Care & Public Health, Faculty of Medicine, School of Public Health, WHO Collaborating Centre, Imperial College London, London, United Kingdom; Cedars-Sinai Heart Institute, UNITED STATES OF AMERICA

## Abstract

**Background and objectives:**

The relationship between increased inflammatory diet patterns and chronic kidney disease (CKD) remains unknown and has not been investigated in large cohorts.

**Methods:**

A total of 154,070 adults registered in UKB database were enrolled and followed, free of CKD and eGFR > 90 mL/min/1.73 m2 at baseline. The energy-adjusted dietary inflammatory index (E-DII) was evaluated using the 24-h recall diet. Cox proportional hazards regression models were used, adjusting for confounders such as demographic indicators, socioeconomic status, and lifestyle factors. Additionally, a subgroup analysis was conducted to investigate the relationship with incident CKD. The nonlinear relationship between E-DII and CKD risk was analyzed using a restricted cubic spline.

**Results:**

During a median follow-up of 11.4 years, 3402 (2.21%) cases occurred. After adjustment for all potential confounders, a higher E-DII was associated with an increased risk of incident CKD (HR for 1 unit increment: 1.05 (1.00–1.10), p = 0.034). According to the result of the restricted cubic spline, when E-DII > 1.857, the risk of early-stage CKD would increase significantly.

**Conclusions:**

A higher pro-inflammatory diet was associated with an increased risk of early-stage CKD in the fully adjusted model. An anti-inflammatory diet may serve as a potential preventive strategy for early-stage CKD, although causal inference cannot be established from this observational study.

## Introduction

Chronic kidney disease (CKD) is a progressive disease with high morbidity and mortality. There is a substantial global burden of CKD [1]. The number of CKD patients worldwide in 2017 was 697.5 million, with 1.2 million deaths each year attributed to CKD [2]. Concurrently, the progression of CKD is frequently accompanied by the onset of cardiovascular diseases (CVD), which has a further detrimental effect on the prognosis for CKD patients. A substantial body of research has indicated that the risk of cardiac arrest is significantly elevated, particularly among dialysis patients in the advanced stages of CKD [3,4]. Therefore, CKD is estimated to become the fifth leading cause of death worldwide by 2040 [5]. There is emerging evidence that continuous low-grade chronic systemic inflammation plays a significant role in the pathogenesis of early-stage CKD [6]. Some inflammatory biomarkers, C-creative protein (CRP), TNF-R1, and TNF-a, have been associated with incident CKD [7,8]. During a 28-year follow-up study, soluble tumor necrosis factor receptors 1 and 2 (sTNFR-1/2) were associated with CKD in patients with type 1 diabetes mellitus [9].

Studies have demonstrated that unhealthy diets contribute to the development of CKD. Regulating systemic circulating inflammatory factors with a healthy diet pattern can be a viable strategy to prevent CKD [10]. For instance, a whole food plant-based diet may reduce glomerular hyperfiltration and protect kidney function by containing anti-inflammatory properties [11]. Dietary pattern accounts for the potential interplay of nutrients in whole diets compared to individual nutrients. Therefore, the relationship between dietary patterns and disease is often investigated [12]. It is unclear whether long-term pro-inflammatory diets are associated with CKD. Since most dietary patterns were not designed to comprehensively explain their potential inflammatory effects, it is unclear whether long-term pro-inflammatory diets are associated with incident CKD. The Dietary inflammatory index (DII) was designed based on concentrations of IL-6, TNFα-R2 and CRP in the literature, it is the first comprehensive indicator to translate individual food intake into an overall inflammatory potential [13]. Various dietary patterns with a higher pro-inflammatory potential have been found to be associated with an increased risk of chronic cardiovascular disease. Limiting the pro-inflammatory potential of the diet may be a viable method for CVD prevention [14]. Studies have shown that a pro-inflammatory diet is associated with decreased kidney function [15–17]. The relationship between inflammatory diets and incident CKD remains unknown and it has not been investigated in a large cohort study. In this study, our objective was to investigate the association between E-DII and incident CKD. The relationship between E-DII and systemic inflammation was further evaluated.

## Methods

### Study population

The UK Biobank study is a prospective population-based cohort with more than 500,000 participants aged 40–70, with available data and open access (https://www.ukbiobank.ac.uk/). The study design has been described in detail [18]. In UKB study, demographic characteristics, sociodemographic status, behaviors, and renal function were collected.

**Fig 1:**
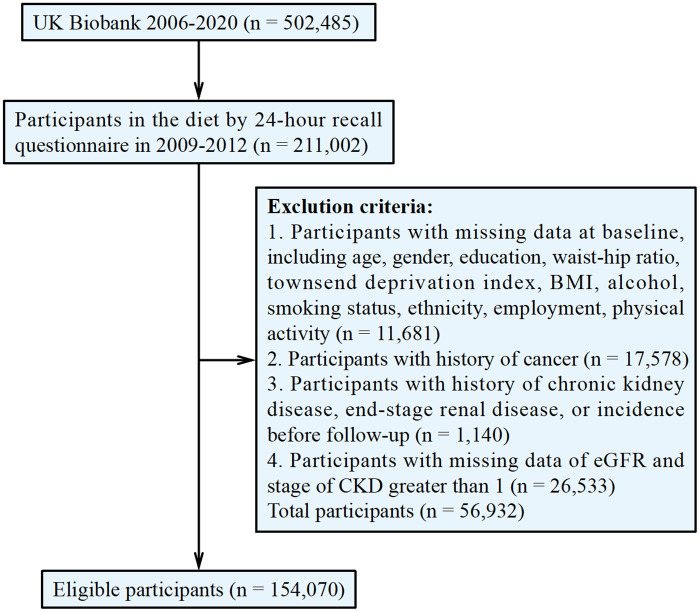
Study flow diagram.

Of these participants, 211,002 completed the 24-hour recall dietary questionnaire in 2009–2012. Exclusion criteria: 1. Participants with missing data at baseline, including age, gender, education, waist-hip ratio, Townsend deprivation index, BMI, alcohol, smoking status, ethnicity, employment, physical activity. 2. Participants with history of cancer. 3. Participants with history of chronic kidney disease, end-stage renal disease, or incidence before follow-up. 4. Participants with missing data of eGFR and stage of CKD greater than 1. After the exclusion of 56,932 participants, the remaining 154,070 individuals were enrolled (Fig 1).

### Calculation of the energy-adjusted dietary inflammatory index

From 2009–2012, the participants completed an online 24-h dietary recall questionnaire, including the first assessment (April 2009 to September 2010), online cycle 1 (February 2011 to April 2011), online cycle 2 (June 2011 to September 2011), online cycle 3 (October 2011 to December 2011), online cycle 4 (April 2012 to June 2012). Volunteers completed online dietary questionnaires after completing the informed consent process. E-DII was previously calculated based on the literature [13,19]. The total E-DII score for each individual is calculated by normalizing a predetermined intake of 45 foods to the central proportions, multiplying them by the respective weights of the inflammatory effect, and summarizing them. The average intake of each parameter was adjusted by 1000 kcal to control the effect of energy consumption on the nutrient intake. A higher E-DII positive score indicates pro-inflammatory diets, and a lower E-DII negative score indicates anti-inflammatory diets [13]. A total of 29 food parameters are available in the UKB database that include saturated fat, total fat, energy, cholesterol, vitamin B12, carbohydrate, protein, MUFA, niacin, riboflavin, caffeine, folic acid, Fe, Se, Mg, niacin, thiamin, Vitamin A, Vitamin C, Vitamin D, Vitamin E, fiber, Zn, green/black tea, garlic, alcohol, Vitamin B6, β-Carotene, and, onion. Studies showed that predictive power was not affected when less than 30 dietary parameters were used. E-DII was calculated without considering the outcome [15,20].

### Assessment of CKD and other covariates

According to the International Classification of Diseases (ICD10) code from first in-patient diagnosis. Early-stage CKD was defined as stage G1-3, and moderate to late-stage CKD was defined as stage G4-5. The estimated glomerular filtration rate (eGFR) was calculated from creatinine and cystatin C using the Modification of Diet in Renal Disease (MDRD) equation. The CKD was graded based on the guideline [21], eGFR stage G1: eGFR ≥ 90 ml/min/1.73m^2^. Stage G2: 60–89 ml/min/1.73m^2^. Stage G3a: 45–59 ml/min/1.73m^2^. Stage G3b: 30–44 ml/min/1.73m^2^. Stage G4: 15–29 ml/min/1.73m^2^. Stage G5: < 15 ml/min/1.73m^2^.

Demographic indicators, socioeconomic status, and lifestyle at baseline, including age, biological gender, race, Townsend deprivation index, body mass index (BMI), education, occupation, smoking, alcohol consumption, frequency of physical activity (>10 mins/week), sleep duration, waist-to-hip ratio (WHratio), creatinine, cystatin C, Self-reported hypertension, CKD, diabetes, and heart disease were collected from participants at baseline. Angiotensin-converting enzyme inhibitors (ACEIs), statins, insulin, metformin, vitamin, and micronutrient supplements were also recorded. The systemic inflammation index (SII) was constructed by the formula: platelet count*neutrophil count/lymphocyte count.

### Statistical analysis

We calculated E-DII scores for five cycles and averaged by the number of attended records. Quartiles of E-DII scores were computed using COX proportional risk regression analysis to calculate hazard ratios (HR) and 95% confidence intervals (CIs) of CKD risk. The median of each quartile was assigned to quantify a linear trend. Potential confounders were screened by univariate COX analysis (S3 Table) (*p* < 0.05). Model 1 adjusted for age and gender. Model 2 added education, employment, smoking status, alcohol consumption, frequency of physical activity (> 10 mins/week), BMI, sleep duration, and waist-to-hip ratio to Model 1. Model 3 added a history of hypertension, diabetes, chronic heart disease, ACEIs, statins, insulin, and metformin to Model 2. The interaction effect between subgroups was examined in the stratification analysis, adjusting for potential confounders in model 3. Furthermore, restricted cubic splines were used to assess the nonlinear relationship between E-DII and incident CKD, adjusting for all cofounders in Model 3. The Spearman correlation coefficients between E-DII and inflammatory indicators and renal function were calculated. All statistical analyses were performed using R for Windows, version 4.1.2., and Stata, version 16. A two-sided *p*-value of less than 0.05 was considered statistically significant.

## Results

### Baseline characteristics of the study population

A total of 154,070 individuals participated in the study. Subjects were divided into four E-DII quartiles, including 1^st^ quartile (0.75 ± 0.47), 2^nd^ quartile (1.58 ± 0.16), 3^rd^ quartile (2.14 ± 0.17), 4^th^ quartile (3.03 ± 0.49). Those in the highest quartile of E-DII were more likely to be male, non-white, lower school graduates, lower Townsend deprivation index, in paid employment, current smokers, higher BMI, and less likely to drink daily than those in the lowest quartiles (all *p* < 0.05). Those in the highest quartile of E-DII were more likely to have hypertension, diabetes, chronic heart disease, ACEIs, β-blocker, statins, insulin, metformin, and vitamin supplement use, and less likely to have mineral supplements than those in the lowest quartiles (all *p* < 0.05). The details are shown in Table 1.

**Table 1:** Baseline characteristics of participants in the UKB stratified by the quartile of energy-adjusted dietary inflammatory index.

Characteristics	Quartile 1	Quartile 2	Quartile 3	Quartile 4
N	38517	38518	38518	38517
Age, years	56.15 (7.95)	55.84 (7.92)	55.53 (7.91)	55.17 (8.01)
Gender, male	17001 (44.1)	17517 (45.5)	18478 (48.0)	19152 (49.7)
Ethnicity, white	37112 (96.4)	37134 (96.4)	37022 (96.1)	36526 (94.8)
Townsend deprivation index	−1.74 (2.78)	−1.69 (2.81)	−1.62 (2.85)	−1.42 (2.96)
E-DII	0.75 (0.47)	1.58 (0.16)	2.14 (0.17)	3.03 (0.49)
Education				
Less than high school	11170 (29.0)	11624 (30.2)	12665 (32.9)	14407 (37.4)
High school or equivalent	7074 (18.4)	7182 (18.6)	7178 (18.6)	7543 (19.6)
Prefessional qualifications	1836 (4.8)	1945 (5.0)	1872 (4.9)	1877 (4.9)
College or above	18437 (47.9)	17767 (46.1)	16803 (43.6)	14690 (38.1)
Employment				
Home	2405 (6.2)	2408 (6.3)	2428 (6.3)	2918 (7.6)
In paid employment	23755 (61.7)	24274 (63.0)	24710 (64.2)	24984 (64.9)
Retired	12357 (32.1)	11836 (30.7)	11380 (29.5)	10615 (27.6)
Smoking status				
Never	22611 (58.7)	22264 (57.8)	21860 (56.8)	21002 (54.5)
Former	13336 (34.6)	13566 (35.2)	13642 (35.4)	13687 (35.5)
Current	2570 (6.7)	2688 (7.0)	3016 (7.8)	3828 (9.9)
Alcohol				
Never	2006 (5.2)	2088 (5.4)	2194 (5.7)	2860 (7.4)
<1 times/week	7370 (19.1)	7349 (19.1)	7778 (20.2)	8769 (22.8)
1-2 times/week	9547 (24.8)	9698 (25.2)	9500 (24.7)	9704 (25.2)
3-4 times/week	10352 (26.9)	10204 (26.5)	9911 (25.7)	9017 (23.4)
Daily	9242 (24.0)	9179 (23.8)	9135 (23.7)	8167 (21.2)
Frequency of 10 + min/week				
0-6 times/week	7036 (18.3)	7289 (18.9)	7511 (19.5)	7203 (18.7)
7-9 times/week	8672 (22.5)	8921 (23.2)	8611 (22.4)	8038 (20.9)
10-12 times/week	9254 (24.0)	8979 (23.3)	8955 (23.2)	8509 (22.1)
13-15 times/week	7500 (19.5)	7444 (19.3)	7374 (19.1)	7643 (19.8)
16-21 times/week	6055 (15.7)	5885 (15.3)	6067 (15.8)	7124 (18.5)
Sleep duration, hours	7.17 (0.99)	7.16 (1.02)	7.14 (1.05)	7.10 (1.12)
BMI, kg/m^2^	26.63 (4.49)	26.76 (4.50)	26.96 (4.55)	27.20 (4.71)
WHratio, %	0.86 (0.09)	0.86 (0.09)	0.87 (0.09)	0.87 (0.09)
Hypertension, %	18713 (48.6)	18887 (49.0)	19213 (49.9)	19430 (50.4)
Diabetes, %	1084 (2.8)	1128 (2.9)	1226 (3.2)	1366 (3.5)
Chronic heart disease, %	1203 (3.1)	1249 (3.2)	1330 (3.5)	1463 (3.8)
ACEIs use, %	2997 (7.8)	3156 (8.2)	3269 (8.5)	3292 (8.5)
β-blocker use, %	1874 (4.9)	1982 (5.1)	2049 (5.3)	2111 (5.5)
Statins, %	5008 (13.0)	5271 (13.7)	5373 (13.9)	5463 (14.2)
Insulin, %	171 (0.4)	159 (0.4)	176 (0.5)	206 (0.5)
Metformin, %	687 (1.8)	797 (2.1)	845 (2.2)	865 (2.2)
Vitamin supplement use, %	12401 (32.2)	12452 (32.3)	12426 (32.3)	12561 (32.6)
Mineral supplement use, %	16982 (44.1)	17006 (44.2)	16712 (43.4)	16289 (42.3)

Normally distributed data are expressed as mean and SD, non-normally distributed data are expressed as median and quartiles, the rest are expressed as counts and percentages. E-DII, energy-adjusted dietary inflammatory index. BMI, body mass index. WHratio, waist-to-hip ratio. ACEIs, angiotensin converting enzyme inhibitors.

### Association between the energy-adjusted dietary inflammatory index and incident CKD

During a median follow-up of 11.4 years, a total of 3402 (2.21%) cases were documented. When the outcome was defined as all incident CKD, the HR (95% CI) was 1.06 (1.03–1.10) in model 1 (*p* = 0.002). The HR (95% CI) was 1.01 (0.97–1.05) for model 2 (*p* = 0.6) and 1.00 (0.97–1.04) for model 3 (*p* = 0.86), which were considered not significant. Furthermore, in model 2, those in the 4^th^ quartile of E-DII had an 11% higher risk of CKD compared with those in the 1^st^ quartile, the HR (95% CI) was 1.11 (1.01–1.22), *p* for trend < 0.001. However, no similar associations were observed in the other models.

When the outcome was early-stage CKD, in model 3, after adjusting for all potential confounders, the HR (95% CI) was 1.05 (1.00–1.10), *p* = 0.034. In Model 1, the HR (95% CI) was 1.28 (1.14–1.43), *p* for trend < 0.001. Similarly, in Model 2, the HR (95% CI) comparing the 4th quartile with the 1^st^ quartile was 1.13 (1.01–1.27), *p* for trend = 0.010, those in the 4^th^ quartile of E-DII had a 13% higher risk compared with those in the 1^st^ quartile. A similar trend could be observed for model 3 (*p* for trend = 0.023). Based on the definition of outcome as moderate to late CKD, no statistically significant differences were found (Table 2).

**Table 2:** HR and 95%CI for chronic kidney disease by the quartile of energy-adjusted dietary inflammatory index.

	HR (95%CI)	*p* value	Quartile of average dietary inflammatory index				*p* for trend
			Quartile 1	Quartile 2	Quartile 3	Quartile 4	
			0.87 [0.52-1.10]	1.59 [1.44-1.73]	2.13 [1.99-2.28]	2.90 [2.65-3.28]	
CKD stage G1-G5							
Cases/person-year			854/441283	787/440927	882/440135	879/437589	
Crude	1.02 (0.98-1.06)	0.246	1.00 (ref.)	0.92 (0.84-1.02)	1.04 (0.94-1.14)	1.04 (0.95-1.15)	0.143
Multivariable Model 1	**1.06 (1.03-1.10)**	**0.002**	1.00 (ref.)	0.96 (0.87-1.06)	**1.11 (1.01-1.22)**	**1.15 (1.04-1.26)**	**<0.001**
Multivariable Model 2	1.01 (0.97-1.05)	0.600	1.00 (ref.)	0.94 (0.85-1.03)	1.04 (0.95-1.14)	1.02 (0.92-1.12)	0.373
Multivariable Model 3	1.00 (0.97-1.04)	0.86	1.00 (ref.)	0.92 (0.84-1.02)	1.02 (0.93-1.12)	1.00 (0.91-1.10)	0.591
Early-stage CKD (G1-G3)							
Cases/person-year			580/443806	547/443245	626/442709	608/440109	
Crude	**1.06 (1.01-1.11)**	**0.012**	1.00 (ref.)	0.96 (0.85-1.08)	1.10 (0.99-1.24)	**1.13 (1.01-1.27)**	**0.008**
Multivariable Model 1	**1.11 (1.06-1.16)**	**<0.001**	1.00 (ref.)	1.00 (0.89-1.13)	**1.19 (1.06-1.33)**	**1.28 (1.14-1.43)**	**<0.001**
Multivariable Model 2	**1.06 (1.01-1.10)**	**0.017**	1.00 (ref.)	0.98 (0.87-1.10)	1.11 (0.99-1.25)	**1.13 (1.01-1.27)**	**0.010**
Multivariable Model 3	**1.05 (1.00-1.10)**	**0.034**	1.00 (ref.)	0.97 (0.86-1.09)	1.09 (0.97-1.2 2)	1.11 (0.99-1.25)	**0.023**
Moderate- and end- stage CKD (G4-G5)							
Cases/person-year			389/442727	353/442274	388/441707	393/439047	
Crude	1.02 (0.96-1.07)	0.59	1.00 (ref.)	0.91 (0.79-1.05)	1.00 (0.87-1.15)	1.03 (0.89-1.18)	0.476
Multivariable Model 1	1.05 (0.99-1.11)	0.086	1.00 (ref.)	0.94 (0.82-1.09)	1.06 (0.92-1.22)	1.12 (0.97-1.29)	0.056
Multivariable Model 2	0.99 (0.93-1.04)	0.645	1.00 (ref.)	0.92 (0.79-1.06)	0.98 (0.85-1.13)	0.96 (0.83-1.11)	0.786
Multivariable Model 3	0.98 (0.93-1.04)	0.492	1.00 (ref.)	0.90 (0.78-1.04)	0.96 (0.83-1.10)	0.94 (0.82-1.09)	0.593

Early-stage CKD was defined as stage I-III, moderate to late-stage CKD was defined as stage IV-V. CI, confidence interval; HR, hazard ratio. Multivariable Model 1, adjusted for age and gender. Multivariable Model 2, adjusted for age and gender education, employment, smoking status, alcohol consumption, frequency of physical activity (> 10 mins/week), BMI, sleep duration, and WHratio. Multivariable Model 3, adjusted for age and gender education, employment, smoking status, alcohol consumption, frequency of physical activity (> 10 mins/week), BMI, sleep duration, WHratio, history of hypertension, diabetes, chronic heart disease, ACEIs, statins, insulin, and metformin.

### The association between energy-adjusted dietary inflammatory index, inflammation indicators, and risk of CKD

**Fig 2:**
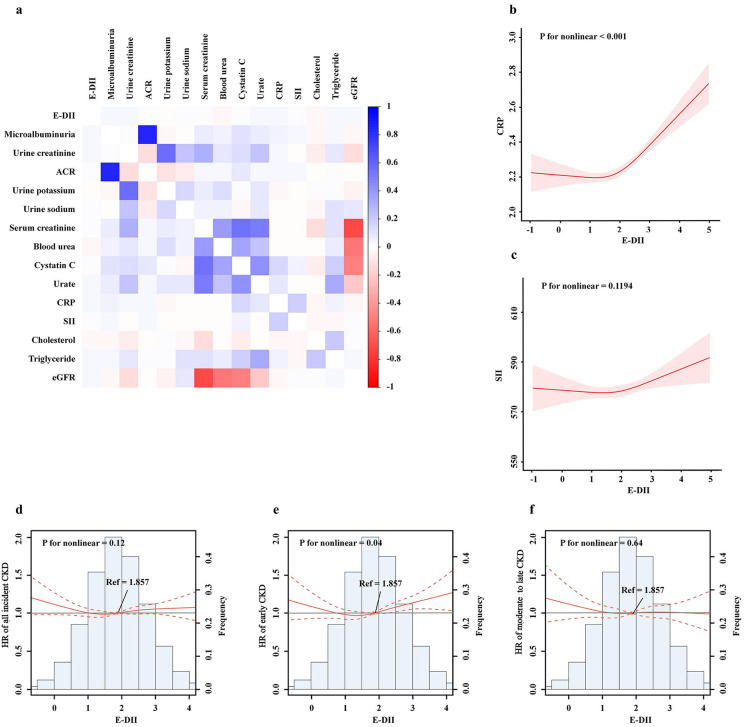
The association between the energy-adjusted dietary inflammatory index and incident CKD. **a)** Heatmap of kidney function, inflammatory indicators and the DII score.Restricted cubic splines of E-DII with **b)** CRP and **c)** SII. The dose-response relationship of the E-DII with **d)** all incident CKD, **e)** early-stage CKD and f) moderate to late CKD.

The association between the E-DII score, inflammation indicators, renal function, and blood lipids was analyzed. We found weak correlations between E-DII and other markers (Fig 2a). The nonlinear relationship between the E-DII score with inflammation indicators was analyzed. It showed that the E-DII score was positively nonlinear associated with CRP (*p* for nonlinear < 0.001, Fig 2b). The SII score also showed the same positive trend, although insignificant (*p* for nonlinear = 0.1194, Fig 2c).

Furthermore, restricted cubic spline models were used to flexibly explore nonlinear relations between the E-DII score and CKD risk (Fig 2d–f). There was a dose-response relationship between the E-DII score and early-stage CKD. Upon reaching 1.857 on the E-DII score, the risk of early-stage CKD increased until reaching the maximum HR (*p* for nonlinear = 0.04). When the E-DII score ≤ 1.857 was taken as a reference group, the risk of early-stage CKD was 1.12 (1.03–1.21), *p* < 0.001. A significant nonlinear relationship was not identified for moderate to late CKD and all incident CKD.

### Stratified analyses of the dietary inflammatory index for association with the risk of CKD

**Fig 3:**
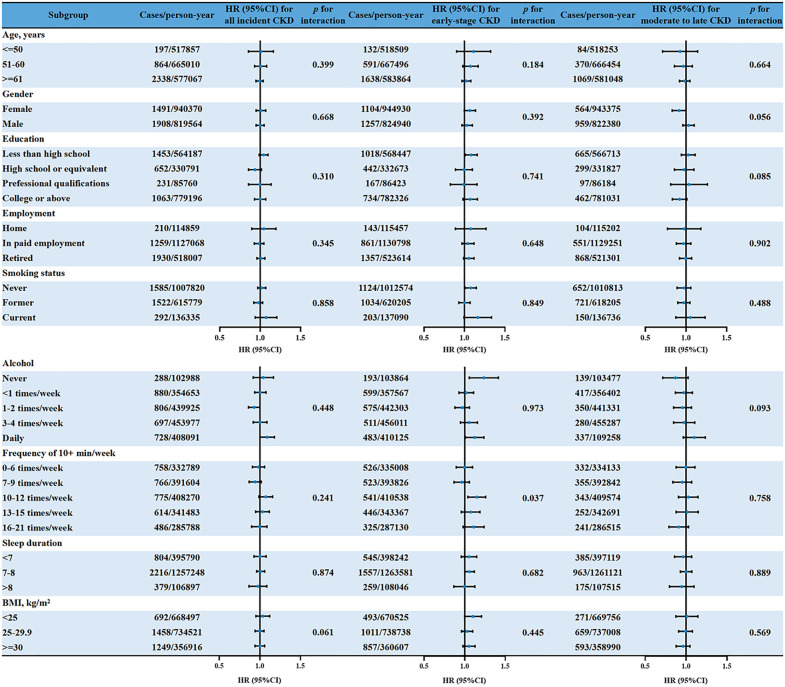
Subgroup analysis and HR (95% CI) for incident CKD associated with energy-adjusted dietary inflammatory index. Adjusted for age and gender education, employment, smoking status, alcohol consumption, frequency of physical activity (> 10 mins/week), BMI, sleep duration, WHratio, history of hypertension, diabetes, chronic heart disease, ACEIs, statins, insulin, and metformin.

All potential confounders were adjusted in the stratified analysis. When the outcome was defined as all incident CKD and moderate to late CKD, the association was generally consistent across all subgroups, stratified by all potential risk confounders (*p* for interaction > 0.05). However, when the outcome was defined as early-stage CKD, there was a difference between subgroups of frequency of physical activity (> 10 mins/week) (P for interaction = 0.037). The association was statistically insignificant in the remaining subgroups (*p* for interaction > 0.05). More details are given in Fig 3 and S1 Table.

## Discussion

In this population-based cohort study, a dietary index based on a literature review was used to assess the inflammatory potential of a diet pattern, and the association between the E-DII score and the risk of CKD was further investigated. An increased risk of CKD was observed in the large UK Biobank cohort for higher E-DII scores. Furthermore, this significant association was only present in early-stage CKD and not in moderate to late CKD and all incident CKD. Compared to individuals with lower E-DII scores, the diet with higher inflammatory potential showed an 11% higher risk in early-stage CKD. Furthermore, the risk of early-stage CKD showed a dose-response relationship with the E-DII score. After the E-DII score reached 1.857, the risk of early-stage CKD started to rise significantly. A stratified analysis revealed differences between subgroups of physical activity. The associations were generally consistent in each subgroup, which indicates the robustness of the results. In summary, a higher pro-inflammatory diet was associated with an increased risk of early-stage CKD, but not with moderate to late CKD and all incident CKD. Our results expand studies that evaluate the E-DII score and the risk of incidence of CKD, and an anti-inflammatory diet can be considered an effective strategy for the primary prevention of CKD [15–17].In this study, it was found that the anti-inflammatory diet demonstrated a stronger association with early-stage CKD, while its association with mid-to-late-stage CKD was weaker.One potential explanation for this finding is that CKD progression is an irreversible process. As the disease progresses, renal structure undergoes changes, and the risk of advanced CKD stages is primarily driven by irreversible renal lesions. By the late stage, renal lesions develop more rapidly, rendering anti-inflammatory dietary interventions less effective. This area merits further exploration in future research. Nevertheless, the findings of the present study suggest that the prevention and control of CKD is of particular importance in the early stages of the disease, and an anti-inflammatory diet still represents a simple and easily achievable strategy.

An increasing body of evidence has shown that chronic inflammation plays an important role in the development of CKD and impaired renal function, especially in the early stage of CKD [22,23]. CKD is a state of chronic systemic inflammation and commonly presents with a constant minor elevation of CRP [24]. The pathogenesis of various forms of CKD is affected by oxidative stress and inflammation, which accelerates their progression. Therefore, inflammation is a potential therapeutic target for CKD prevention and treatment; we believe that diet could be a viable prevention and management option for CKD [10]. Several recent studies have described plant-based diets as beneficial for CKD, but they can also cause malnutrition in patients with CKD [11,25,26]. The inflammatory dietary pattern has been reported to be associated with cardiovascular disease, diabetes, and cancer [14,27–29]. However, there is sparse evidence regarding the association between inflammatory dietary patterns and impaired kidney function in the early stage. Previous studies have investigated the association between pro-inflammatory dietary patterns and systemic inflammation and kidney functions [15–17,30,31]. Our results are consistent with the studies mentioned above. In a cross-sectional study with 2644 women aged 45–75, the association between high DII scores was significantly associated with a reduction of eGFR (p = 0.006) [31]. A cross-sectional study of 1422 older women over 70 years found that the DII score was not linearly associated with the risk of CKD [17]. Moreover, it was observed that a pro-inflammatory diet was associated with the progression of CKD in a cross-sectional study with 221 patients; this study showed that OR_Q3VSQ1_ was 2.12 (1.05–4.26), p for trend = 0.03 [16]. Since most studies used cross-sectional designs, causal associations could not be determined, and these studies included small sample sizes [15–17,31].

There are a few strengths to our study. Firstly, the present study investigated the association between the DII score and CKD as part of a large prospective population-based cohort study. Secondly, since we excluded those with baseline renal insufficiency, the level of evidence could be higher than in cross-sectional studies. Thirdly, subgroup analyses were conducted, and all potential confounders were adjusted carefully, so the reliability of our results increased. Lastly, we proposed a healthy diet with an optimal E-DII score of 1.857 to control potential inflammation.

Our study also had some limitations. The 24 h recall diet was relatively subjective. Underreporting and recall bias cannot be completely avoided. However, based on existing studies of the E-DII we conducted a systematic assessment of dietary inflammation in this study. While more validation in future updates and other populations is planned, this study offers a foundational clu for further research. Second, only 29 of 45 recommended food parameters were used to calculate the E-DII score, resulting in a narrow range of values for E-DII. This could have affected the study results. However, previous studies have shown that reducing the number of food parameters to less than 30 would not affect the objectivity of the results [15,20]. Another potential weakness is that the study was conducted among the UK population, making the results ungeneralizable to other populations. Further clinical trials with more comprehensive and detailed diets and early-stage CKD assessments are required. Different countries or regions should be examined for a more reliable result and smaller errors. Furthermore, using ICD10 codes alone will miss many cases of undiagnosed CKD, and most of the follow-up eGFR data are missing. It is necessary to use more detailed diagnostic criteria in future studies. Finally, we attempted to design a prospective cohort study, excluding patients with a history of CKD at baseline and conducting a long-term follow-up with a median duration of 11.4 years. We made every effort to provide some clues for causal relationships. However, caution is still warranted when interpreting causal relationships. Nevertheless, this study provides valuable insights for future research.

## Conclusions

Our results demonstrated that high pro-inflammatory diets were associated with an increased risk of early-stage CKD in this large prospective cohort study. Our study suggested that an anti-inflammatory diet with an optimal E-DII score could be considered an effective strategy for CKD prevention and treatment before implementing and formulating nutritional policies.

## Supporting information

S1 Table Energy adjusted intakes of food parameters contributing to the Dietary Inflammatory Index (DII) score according to the quartile of dietary inflammatory index. (DOCX)

S2 Table HR and 95%CI for chronic kidney disease according to energy adjusted intakes of food parameters. (DOCX)

S3 Table Univariate analysis of covariates and chronic. (DOCX)

## References

[1] Kalantar-Zadeh K, Jafar TH, Nitsch D, Neuen BL, Perkovic V. Chronic kidney disease. Lancet. 2021;398(10302):786–802. doi: 10.1016/S0140-6736(21)00519-5 34175022

[2] GBD Chronic Kidney Disease Collaboration. Global, regional, and national burden of chronic kidney disease, 1990-2017: A systematic analysis for the Global Burden of Disease Study 2017. Lancet. 2020;395(10225):709–33. doi: 10.1016/S0140-6736(20)30045-3 32061315 PMC7049905

[3] Truyen TTTT, Uy-Evanado A, Chugh H, Reinier K, Charytan DM, Salvucci A, et al. Moderate kidney dysfunction independently increases sudden cardiac arrest risk: A community-based study. J Am Heart Assoc. 2025;14(15):e042307. doi: 10.1161/JAHA.125.042307 40728166 PMC12449910

[4] Marx-Schütt K, Cherney DZI, Jankowski J, Matsushita K, Nardone M, Marx N. Cardiovascular disease in chronic kidney disease. Eur Heart J. 2025;46(23):2148–60. doi: 10.1093/eurheartj/ehaf167 40196891 PMC12167664

[5] Foreman KJ, Marquez N, Dolgert A, Fukutaki K, Fullman N, McGaughey M, et al. Forecasting life expectancy, years of life lost, and all-cause and cause-specific mortality for 250 causes of death: Reference and alternative scenarios for 2016-40 for 195 countries and territories. Lancet. 2018;392(10159):2052–90. doi: 10.1016/S0140-6736(18)31694-5 30340847 PMC6227505

[6] Furman D, Campisi J, Verdin E, Carrera-Bastos P, Targ S, Franceschi C, et al. Chronic inflammation in the etiology of disease across the life span. Nat Med. 2019;25(12):1822–32. doi: 10.1038/s41591-019-0675-0 31806905 PMC7147972

[7] Liu L, Gao B, Wang J, Yang C, Wu S, Wu Y, et al. Clinical significance of single and persistent elevation of serum high-sensitivity C-reactive protein levels for prediction of kidney outcomes in patients with impaired fasting glucose or diabetes mellitus. J Nephrol. 2021;34(4):1179–88. doi: 10.1007/s40620-020-00848-4 32880885

[8] Feng Y-M, Thijs L, Zhang Z-Y, Yang W-Y, Huang Q-F, Wei F-F, et al. Glomerular function in relation to circulating adhesion molecules and inflammation markers in a general population. Nephrol Dial Transplant. 2018;33(3):426–35. doi: 10.1093/ndt/gfx256 28992257 PMC6018976

[9] Baker NL, Hunt KJ, Stevens DR, Jarai G, Rosen GD, Klein RL, et al. Association between inflammatory markers and progression to kidney dysfunction: Examining different assessment windows in patients with type 1 diabetes. Diabetes Care. 2018;41(1):128–35. doi: 10.2337/dc17-0867 29118060 PMC5741153

[10] Mafra D, Borges NA, Lindholm B, Shiels PG, Evenepoel P, Stenvinkel P. Food as medicine: Targeting the uraemic phenotype in chronic kidney disease. Nat Rev Nephrol. 2021;17(3):153–71. doi: 10.1038/s41581-020-00345-8 32963366

[11] Adair KE, Bowden RG. Ameliorating chronic kidney disease using a whole food plant-based diet. Nutrients. 2020;12(4):1007. doi: 10.3390/nu12041007 32268544 PMC7230354

[12] Schulz C-A, Oluwagbemigun K, Nöthlings U. Advances in dietary pattern analysis in nutritional epidemiology. Eur J Nutr. 2021;60(8):4115–30. doi: 10.1007/s00394-021-02545-9 33899149 PMC8572214

[13] Shivappa N, Steck SE, Hurley TG, Hussey JR, Hébert JR. Designing and developing a literature-derived, population-based dietary inflammatory index. Public Health Nutr. 2014;17(8):1689–96. doi: 10.1017/S1368980013002115 23941862 PMC3925198

[14] Li J, Lee DH, Hu J, Tabung FK, Li Y, Bhupathiraju SN, et al. Dietary inflammatory potential and risk of cardiovascular disease among men and women in the u.S. J Am Coll Cardiol. 2020;76(19):2181–93. doi: 10.1016/j.jacc.2020.09.535 33153576 PMC7745775

[15] Mazidi M, Shivappa N, Wirth MD, Hebert JR, Kengne AP. Greater Dietary Inflammatory Index score is associated with higher likelihood of chronic kidney disease. Br J Nutr. 2018;120(2):204–9. doi: 10.1017/S0007114518001071 29947319

[16] Rouhani MH, Najafabadi MM, Surkan PJ, Esmaillzadeh A, Feizi A, Azadbakht L. Dietary inflammatory index and its association with renal function and progression of chronic kidney disease. Clin Nutr ESPEN. 2019;29:237–41. doi: 10.1016/j.clnesp.2018.09.001 30661693

[17] Bondonno NP, Blekkenhorst LC, Bird AL, Lewis JR, Hodgson JM, Shivappa N, et al. Dietary inflammatory index and the aging kidney in older women: A 10-year prospective cohort study. Eur J Nutr. 2020;59(7):3201–11. doi: 10.1007/s00394-019-02160-9 31828473

[18] Sudlow C, Gallacher J, Allen N, Beral V, Burton P, Danesh J, et al. UK biobank: An open access resource for identifying the causes of a wide range of complex diseases of middle and old age. PLoS Med. 2015;12(3):e1001779. doi: 10.1371/journal.pmed.1001779 25826379 PMC4380465

[19] Hébert JR, Shivappa N, Wirth MD, Hussey JR, Hurley TG. Perspective: The dietary inflammatory index (DII)-lessons learned, improvements made, and future directions. Adv Nutr. 2019;10(2):185–95. doi: 10.1093/advances/nmy071 30615051 PMC6416047

[20] Qin Z, Yang Q, Liao R, Su B. The Association Between Dietary Inflammatory index and parathyroid hormone in adults with/without chronic kidney disease. Front Nutr. 2021;8:688369. doi: 10.3389/fnut.2021.688369 34249998 PMC8266995

[21] Stevens PE, Levin A, Kidney Disease: Improving Global Outcomes Chronic Kidney Disease Guideline Development Work Group Members. Evaluation and management of chronic kidney disease: Synopsis of the kidney disease: improving global outcomes 2012 clinical practice guideline. Ann Intern Med. 2013;158(11):825–30. doi: 10.7326/0003-4819-158-11-201306040-00007 23732715

[22] Waters DD, Vogt L. Lipids, inflammation, and chronic kidney disease: A sharp perspective. Kidney Int. 2018;93(4):784–6. doi: 10.1016/j.kint.2017.11.031 29571452

[23] Ebert T, Neytchev O, Witasp A, Kublickiene K, Stenvinkel P, Shiels PG. Inflammation and oxidative stress in chronic kidney disease and dialysis patients. Antioxid Redox Signal. 2021;35(17):1426–48. doi: 10.1089/ars.2020.8184 34006115

[24] Carrero JJ, Stenvinkel P. Inflammation in end-stage renal disease--what have we learned in 10 years?. Semin Dial. 2010;23(5):498–509. doi: 10.1111/j.1525-139X.2010.00784.x 21039875

[25] Kim H, Caulfield LE, Garcia-Larsen V, Steffen LM, Grams ME, Coresh J, et al. Plant-based diets and incident CKD and kidney function. Clin J Am Soc Nephrol. 2019;14(5):682–91. doi: 10.2215/CJN.12391018 31023928 PMC6500948

[26] Carrero JJ, González-Ortiz A, Avesani CM, Bakker SJL, Bellizzi V, Chauveau P, et al. Plant-based diets to manage the risks and complications of chronic kidney disease. Nat Rev Nephrol. 2020;16(9):525–42. doi: 10.1038/s41581-020-0297-2 32528189

[27] Laouali N, Mancini FR, Hajji-Louati M, El Fatouhi D, Balkau B, Boutron-Ruault MC, et al. Dietary inflammatory index and type 2 diabetes risk in a prospective cohort of 70,991 women followed for 20 years: The mediating role of bmi. Diabetologia. 2019;62(12):2222–32. doi: 10.1007/s00125-019-04972-0 31396661

[28] Hariharan R, Odjidja EN, Scott D, Shivappa N, Hébert JR, Hodge A, et al. The dietary inflammatory index, obesity, type 2 diabetes, and cardiovascular risk factors and diseases. Obes Rev. 2022;23(1):e13349. doi: 10.1111/obr.13349 34708499

[29] Fu BC, Tabung FK, Pernar CH, Wang W, Gonzalez-Feliciano AG, Chowdhury-Paulino IM, et al. Insulinemic and inflammatory dietary patterns and risk of prostate cancer. Eur Urol. 2021;79(3):405–12. doi: 10.1016/j.eururo.2020.12.030 33422354 PMC7887049

[30] Xu H, Sjögren P, Ärnlöv J, Banerjee T, Cederholm T, Risérus U, et al. A proinflammatory diet is associated with systemic inflammation and reduced kidney function in elderly adults. J Nutr. 2015;145(4):729–35. doi: 10.3945/jn.114.205187 25833776

[31] Lin M, Shivappa N, Hébert JR, Huang H, Cai L, Liang J, et al. Dietary inflammatory index and cardiorenal function in women with diabetes and prediabetes. Nutr Metab Cardiovasc Dis. 2021;31(8):2319–27. doi: 10.1016/j.numecd.2021.05.011 34154885

