# Supplementary material for: Dietary inflammatory index in relation to incident CKD: A prospective study of UK Biobank participants
Source: PLoS One. 2026 Feb 20;21(2):e0341502. doi: 10.1371/journal.pone.0341502 (PMC12923052; doi:10.1371/journal.pone.0341502)
Supplement: S1 Table — (DOCX) [file pone.0341502.s001.docx]

**Supplemental Table 1. Energy adjusted intakes of food parameters contributing to the Dietary Inflammatory Index (DII) score according to the quartile of dietary inflammatory index.**

| Food parameters | Quartile of dietary inflammatory index | | | | *p* value |
| --- | --- | --- | --- | --- | --- |
|  | Q1 | Q2 | Q3 | Q4 |  |
|  | n=38517 | n=38518 | n=38518 | n=38517 |  |
| Energy (MJ/d) | 37.6 ± 10.2 | 36.9 ± 10.0 | 36.8 ± 10.9 | 37.6 ± 14.1 | <0.001 |
| Total fat (g/MJ) | 2.25 ± 0.45 | 2.09 ± 0.42 | 2.06 ± 0.42 | 2.03 ± 0.44 | <0.001 |
| Saturated fat (g/MJ) | 0.84 ± 0.24 | 0.80 ± 0.21 | 0.79 ± 0.20 | 0.77 ± 0.22 | <0.001 |
| MUFAs (g/MJ) | 0.59 ± 0.29 | 0.61 ± 0.29 | 0.61 ± 0.31 | 0.62 ± 0.36 | <0.001 |
| PUFAs (g/MJ) | 0.39 ± 0.13 | 0.38 ± 0.14 | 0.38 ± 0.14 | 0.38 ± 0.17 | <0.001 |
| Cholesterol (mg/MJ) | 8.49 ± 10.55 | 8.11 ± 9.54 | 8.10 ± 10.06 | 8.71 ± 12.78 | <0.001 |
| Protein (g/MJ) | 2.25 ± 0.50 | 2.27 ± 0.48 | 2.28 ± 0.51 | 2.28 ± 0.58 | <0.001 |
| Carbohydrate (g/MJ) | 6.83 ±1.15 | 6.88 ± 1.15 | 6.90 ± 1.19 | 6.99 ± 1.33 | <0.001 |
| Fibre (g/MJ) | 0.47 ± 0.15 | 0.45 ± 1.47 | 0.44 ± 0.16 | 0.45 ± 0.21 | <0.001 |
| Alcohol (g/MJ) | 0.58 ± 0.47 | 0.62 ± 0.51 | 0.68 ± 0.56 | 0.77 ± 0.64 | <0.001 |
| Retinol (mg/MJ) | 9.53 ± 4.37 | 8.70 ± 3.83 | 8.32 ± 3.74 | 8.02 ± 4.21 | <0.001 |
| Β-Carotene (µg/MJ) | 91.58 ± 63.96 | 86.18 ± 67.18 | 82.20 ± 73.22 | 89.34 ± 98.24 | <0.001 |
| Thiamin (mg/MJ) | 0.025 ± 0.010 | 0.025 ± 0.010 | 0.026 ± 0.011 | 0.026 ± 0.013 | <0.001 |
| Riboflavin (µg/MJ) | 0.034 ± 0.011 | 0.033 ± 0.011 | 0.033 ± 0.011 | 0.033 ± 0.013 | <0.001 |
| Niacin (mg/MJ) | 0.758 ± 0.242 | 0.758 ± 0.239 | 0.757 ± 0.253 | 0.765 ± 0.292 | <0.001 |
| Folate (µg/MJ) | 8.28 ± 2.64 | 8.34 ± 2.59 | 8.41 ± 2.77 | 8.65 ± 4.00 | <0.001 |
| Vitamin B6 (mg/MJ) | 0.059 ± 0.018 | 0.060 ± 0.017 | 0.061 ± 0.017 | 0.061 ± 0.020 | <0.001 |
| Vitamin B12 (µg/MJ) | 0.187 ± 0.127 | 0.185 ± 0.122 | 0.182 ± 0.122 | 0.175 ± 0.132 | <0.001 |
| Vitamin C (mg/MJ) | 4.05 ± 2.49 | 4.23 ± 2.68 | 4.27 ± 2.90 | 4.42 ± 3.71 | <0.001 |
| Vitamin D (µg/MJ) | 0.087 ± 0.078 | 0.082 ± 0.074 | 0.077 ± 0.073 | 0.072 ± 0.080 | <0.001 |
| Vitamin E (mg/MJ) | 0.260 ± 0.20 | 0.252 ± 0.083 | 0.244 ± 0.089 | 0.244 ± 0.119 | <0.001 |
| Iron (mg/MJ) | 0.379 ± 0.090 | 0.379 ± 0.086 | 0.375 ± 0.087 | 0.373 ± 0.108 | <0.001 |
| Magnesium (mg/MJ) | 9.43 ± 1.90 | 9.55 ± 1.72 | 9.59 ± 1.76 | 9.81 ± 5.81 | <0.001 |
| Zinc (mg/MJ) | 0.223 ± 0.083 | 0.222 ± 0.081 | 0.221 ± 0.087 | 0.224 ± 0.101 | <0.001 |
| Se (µg/MJ) | 3.97 ± 3.74 | 3.90 ± 3.46 | 3.79 ± 3.42 | 3.73 ± 3.65 | <0.001 |
| Garlic (mg/MJ) | 0.049 ± 0.052 | 0.045 ± 0.049 | 0.046 ± 0.056 | 0.059 ± 0.067 | <0.001 |
| Onion (mg/MJ) | 0.94 ± 0.87 | 0.90 ± 0.86 | 0.98 ± 0.90 | 1.28 ± 1.06 | <0.001 |
| Green/black tea (g/MJ) | 0.069 ± 0.043 | 0.079 ± 0.048 | 0.089 ± 0.052 | 0.111 ± 0.464 | <0.001 |
| Caffeine (g/MJ) | 0.005 ± 0.004 | 0.005 ± 0.004 | 0.005 ± 0.004 | 0.006 ± 0.045 | <0.001 |

Data expressed as mean ± SD unless otherwise stated. *p* values for comparisons between the quartile of dietary inflammatory index were derived by ANOVA or Kruskal-Wallis H test where appropriate. DII, Dietary Inflammatory Index; MUFA, Monounsaturated fatty acid; PUFA, polyunsaturated fatty acid.
