# Supplementary material for: Dietary inflammatory index in relation to incident CKD: A prospective study of UK Biobank participants
Source: PLoS One. 2026 Feb 20;21(2):e0341502. doi: 10.1371/journal.pone.0341502 (PMC12923052; doi:10.1371/journal.pone.0341502)
Supplement: S2 Table — (DOCX) [file pone.0341502.s002.docx]

**Supplemental Table 2. HR and 95%CI for chronic kidney disease according to energy adjusted intakes of food parameters**

| Food parameters | CKD stage I-V | | | | Early-stage CKD (I-III) | | | | Moderate- and advanced- stage CKD (IV-V) | | | |
| --- | --- | --- | --- | --- | --- | --- | --- | --- | --- | --- | --- | --- |
|  | β coefficient | HR | 95%CI | *p* value | β coefficient | HR | 95%CI | *p* value | β coefficient | HR | 95%CI | *p* value |
| Energy (MJ/d) | -0.0003 | 1.000 | 0.997-1.003 | 0.868 | -0.00001 | 1.000 | 1.000 | 0.106 | 0.00001 | 1.000 | 1.000 | 0.236 |
| Total fat (g) | 0.0006 | 1.001 | 1.000-1.002 | 0.284 | -0.0005 | 1.000 | 0.998-1.001 | 0.475 | 0.0014 | 1.001 | 1.000-1.003 | 0.087 |
| Saturated fat (g) | 0.002 | 1.002 | 0.999-1.004 | 0.140 | -0.0008 | 0.999 | 0.996-1.002 | 0.575 | 0.0039 | 1.004 | 1.000-1.007 | 0.034 |
| MUFAs (g) | 0.008 | 1.008 | 1.006-1.010 | <0.001 | 0.0065 | 1.007 | 1.003-1.010 | <0.001 | 0.0092 | 1.009 | 1.006-1.013 | <0.001 |
| PUFAs (g) | 0.004 | 1.004 | 0.999-1.008 | <0.001 | 0.0012 | 1.001 | 0.996-1.007 | 0.667 | 0.0055 | 1.006 | 0.999-1.012 | 0.103 |
| Cholesterol (mg) | 0.0003 | 1.000 | 1.0002-1.0003 | <0.001 | 0.0006 | 1.001 | 1.000-1.001 | <0.001 | 0.0003 | 1.000 | 1.0003-1.0004 | <0.001 |
| Protein (g) | 0.001 | 1.001 | 1.000-1.002 | 0.003 | 0.0005 | 1.000 | 0.999-1.002 | 0.534 | 0.0016 | 1.002 | 1.000-1.003 | 0.093 |
| Carbohydrate (g) | 0.0003 | 1.000 | 1.000-1.001 | 0.131 | 0.0004 | 1.000 | 1.000 | 0.979 | 0.0007 | 1.000 | 1.0001-1.0011 | 0.011 |
| Fibre (g) | -0.0003 | 1.000 | 0.995-1.005 | 0.893 | -0.0034 | 0.997 | 0.990-1.003 | 0.283 | -0.0011 | 0.999 | 0.991-1.007 | 0.780 |
| Alcohol (g) | -0.004 | 0.996 | 0.994-0.998 | 0.001 | -0.0031 | 0.997 | 0.994-0.999 | 0.017 | -0.0032 | 0.997 | 0.994-1.000 | 0.049 |
| Retinol (mg) | 0.0001 | 1.000 | 1.000-1.0003 | 0.316 | -0.00002 | 1.000 | 1.000 | 0.839 | 0.0001 | 1.000 | 1.000 | 0.393 |
| Β-Carotene (µg) | -0.0003 | 1.000 | 0.999-1.000 | 0.518 | -0.00001 | 1.000 | 1.000 | 0.638 | -0.00003 | 1.000 | 1.000 | 0.007 |
| Thiamin (mg) | 0.101 | 1.106 | 1.031-1.187 | 0.005 | 0.0605 | 1.062 | 0.974-1.159 | 0.174 | 0.1875 | 1.206 | 1.090-1.334 | <0.001 |
| Riboflavin (µg) | 0.193 | 1.213 | 1.135-1.297 | <0.001 | 0.1548 | 1.167 | 1.074-1.269 | <0.001 | 0.263 | 1.301 | 1.183-1.430 | <0.001 |
| Niacin (mg) | 0.010 | 1.010 | 1.007-1.013 | <0.001 | 0.0081 | 1.008 | 1.005-1.012 | <0.001 | 0.0136 | 1.014 | 1.010-1.018 | <0.001 |
| Folate (µg) | 0.0002 | 1.000 | 1.000-1.001 | 0.079 | 0.0002 | 1.000 | 1.000 | 0.220 | 0.0003 | 1.000 | 1.000 | 0.161 |
| Vitamin B6 (mg) | 0.0792 | 1.082 | 1.036-1.131 | <0.001 | 0.0683 | 1.071 | 1.014-1.131 | 0.014 | 0.0902 | 1.094 | 1.025-1.168 | 0.007 |
| Vitamin B12 (µg) | 0.007 | 1.007 | 1.000-1.014 | 0.043 | 0.0064 | 1.006 | 0.998-1.015 | 0.143 | 0.0136 | 1.014 | 1.003-1.024 | 0.008 |
| Vitamin C (mg) | -0.0005 | 0.999 | 0.999-1.000 | 0.003 | -0.0006 | 0.999 | 0.999-1.000 | 0.003 | -0.0006 | 0.999 | 0.9989-0.9999 | 0.016 |
| Vitamin D (µg) | 0.004 | 1.004 | 0.992-1.017 | 0.489 | 0.0030 | 1.003 | 0.988-1.018 | 0.687 | 0.0047 | 1.005 | 0.987-1.023 | 0.611 |
| Vitamin E (mg) | -0.020 | 0.980 | 0.973-0.988 | <0.001 | -0.0255 | 0.975 | 0.965-0.984 | <0.001 | -0.0221 | 0.978 | 0.967-0.990 | <0.001 |
| Iron (mg) | -0.009 | 0.991 | 0.983-0.999 | 0.020 | -0.0167 | 0.983 | 0.974-0.993 | 0.001 | -0.0055 | 0.994 | 0.983-1.006 | 0.342 |
| Magnesium (mg) | -0.0008 | 0.999 | 0.999-1.000 | <0.001 | -0.0011 | 0.999 | 0.998-0.999 | <0.001 | -0.0007 | 0.999 | 0.999-1.000 | 0.004 |
| Zinc (mg) | 0.017 | 1.017 | 1.007-1.026 | <0.001 | 0.0105 | 1.011 | 0.999-1.022 | 0.076 | 0.0262 | 1.027 | 1.013-1.040 | <0.001 |
| Se (µg) | 0.0001 | 1.000 | 0.9998-1.0003 | 0.584 | -0.00007 | 1.000 | 1.000 | 0.700 | 0.0002 | 1.000 | 1.000 | 0.369 |
| Garlic (mg) | -0.007 | 0.993 | 0.948-1.039 | 0.753 | -0.0206 | 0.980 | 0.926-1.037 | 0.475 | 0.0239 | 1.024 | 0.958-1.096 | 0.486 |
| Onion (mg) | 0.001 | 1.001 | 0.999-1.003 | 0.170 | 0.0021 | 1.002 | 1.000-1.004 | 0.047 | 0.0015 | 1.002 | 0.999-1.004 | 0.258 |
| Green/black tea (g) | 0.049 | 1.051 | 1.025-1.077 | <0.001 | 0.0616 | 1.064 | 1.033-1.095 | <0.001 | 0.0545 | 1.056 | 1.018-1.096 | 0.004 |
| Caffeine (g) | -1.024 | 0.359 | 0.255-0.505 | <0.001 | -1.310 | 0.270 | 0.178-0.409 | <0.001 | -0.8998 | 0.407 | 0.245-0.675 | <0.001 |
