# Supplementary material for: Dietary inflammatory index in relation to incident CKD: A prospective study of UK Biobank participants
Source: PLoS One. 2026 Feb 20;21(2):e0341502. doi: 10.1371/journal.pone.0341502 (PMC12923052; doi:10.1371/journal.pone.0341502)
Supplement: S3 Table — (DOCX) [file pone.0341502.s003.docx]

| **Supplementary Table3. Univariate analysis of covariates and chronic kidney disease.** | | | | |
| --- | --- | --- | --- | --- |
| **Variable** | **HR** | **Lower_95** | **Upper_95** | **p.value** |
| Age, years | 1.14 | 1.14 | 1.15 | <0.001 |
| Gender, male | 1.35 | 1.25 | 1.46 | <0.001 |
| Ethnicity, white | 0.965 | 0.783 | 1.19 | 0.736 |
| Townsend deprivation index | 1 | 0.988 | 1.02 | 0.814 |
| Education |  |  |  | <0.001(p for trend) |
| Less than high school | ref |  |  |  |
| High school or equivalent | 0.746 | 0.669 | 0.832 | <0.001 |
| Prefessional qualifications | 1.06 | 0.902 | 1.25 | 0.477 |
| College or above | 0.516 | 0.47 | 0.566 | <0.001 |
| Employment |  |  |  | <0.001(p for trend) |
| Home | ref |  |  |  |
| In paid employment | 3.52 | 3.24 | 3.82 | <0.001 |
| Retired | 1.67 | 1.4 | 1.98 | <0.001 |
| Smoking status |  |  |  | <0.001(p for trend) |
| Never | ref |  |  |  |
| Former | 1.53 | 1.41 | 1.66 | <0.001 |
| Current | 1.41 | 1.22 | 1.63 | <0.001 |
| Alcohol |  |  |  | <0.001(p for trend) |
| Never | ref |  |  |  |
| <1 times/week | 0.871 | 0.743 | 1.02 | 0.0871 |
| 1-2 times/week | 0.681 | 0.581 | 0.798 | <0.001 |
| 3-4 times/week | 0.586 | 0.499 | 0.689 | <0.001 |
| Daily | 0.629 | 0.535 | 0.74 | <0.001 |
| Frequency of 10+ min/week |  |  |  | <0.001(p for trend) |
| 0-6 times/week | ref |  |  |  |
| 7-9 times/week | 0.838 | 0.745 | 0.943 | 0.003 |
| 10-12 times/week | 0.83 | 0.738 | 0.933 | 0.002 |
| 13-15 times/week | 0.823 | 0.728 | 0.931 | 0.002 |
| 16-21 times/week | 0.727 | 0.636 | 0.832 | <0.001 |
| Sleep duration, hours | 1.09 | 1.04 | 1.13 | <0.001 |
| BMI | 1.09 | 1.08 | 1.1 | <0.001 |
| WHratio | 1.62 | 1.56 | 1.68 | <0.001 |
| Diabetes | 4.54 | 4.04 | 5.1 | <0.001 |
| Hypertension | 3.21 | 2.93 | 3.52 | <0.001 |
| Chronic heart disease | 5.34 | 4.76 | 5.99 | <0.001 |
| ACEIs use | 4.23 | 3.86 | 4.62 | <0.001 |
| Statins | 4.13 | 3.81 | 4.48 | <0.001 |
| Insulin | 7.12 | 5.62 | 9.03 | <0.001 |
| Metformin | 5.09 | 4.44 | 5.85 | <0.001 |
| Vitamin/Mineral Supplement use | 1.06 | 0.975 | 1.14 | 0.182 |
